# Cholera

**Published:** 1849-07

**Authors:** 


### ARTICLE VI.

### THE CHOLERA.

This epidemic now appears to be prevalent in most of the cities, and many of the small towns, in the south-west and West, and is spreading among the great cities of the East.

A remarkable feature of the present epidemic is that it spreads from West, Eastwardly, while in its previous appearence in the country, the reverse was the course it pursued.

We shall in the next number give facts in this connection, from which the opinion of its spreading by communication from one individual to another, will be fairly inferable if not anerresistible conclusion.

In Chicago it has gradually spread from one case that occurred on board a boat from St. Louis, and a family of English emigrants, recently arrived, to most parts of the city; yet the degree of mortality has been very moderate compared with other cities in the West.

We learn from Dr. L. D. Boone, City Health Officer and Physician to the Cholera Hospital, that the number of deaths from cholera during the two months of its prevalence here, has been 154. Also, that the number of cases that have occurred, including those with premonitory diarrhœa, rice water discharges &c., has been, at least, one thousand. The mortality has been mostly among foreigners.

We have had, thus far, a pretty uniform mortality during the two months, no day having more than nine deaths, and the average being about two and a half per diem.

We observed that the very warm weather during the last week of June increased the mortality to the maximum above stated, while the cool weather since has reduced it to the average.

The treatment pursued by the physicians varies to some extent, especially in the early stages, but after its full development, the use of anodyne and astringent remedies given per anum and per os to check the diarrhœa, are resorted to generally; with tonics and stimulants, variously selected to suit the case, generally partaking largely of quinine to restore the tone and activity to the system. Calomel is relied upon by some, but is given with other remedies.

The sulphur treatment for the premonitory stage of the disease, is relied upon by some of our physicians, a full account of which will be found on the succeeding pages of this Journal, by our late colleague of the Editorial Department, Prof. Herrick. E.

